# Invadopodia Structure in 3D Environment Resolved by Near-Infrared Branding Protocol Combining Correlative Confocal and FIB-SEM Microscopy

**DOI:** 10.3390/ijms22157805

**Published:** 2021-07-21

**Authors:** Markéta Dalecká, Ján Sabó, Lenka Backová, Daniel Rösel, Jan Brábek, Aleš Benda, Ondřej Tolde

**Affiliations:** 1 Imaging Methods Core Facility at BIOCEV, Faculty of Science, Charles University, Průmyslová 595, 25242 Vestec u Prahy, Czech Republic; marketa.dalecka@natur.cuni.cz (M.D.); jan.sabo@natur.cuni.cz (J.S.); lenka.backova@natur.cuni.cz (L.B.); 2 Department of Cell Biology, Charles University, Viničná 7, 12800 Prague, Czech Republic; daniel.rosel@natur.cuni.cz (D.R.); jan.brabek@natur.cuni.cz (J.B.); 3 Department of Physical Chemistry, Charles University, Hlavova 8, 12800 Prague, Czech Republic; 4 Biotechnology and Biomedicine Centre of the Academy of Sciences and Charles University (BIOCEV), Průmyslová 595, 25242 Vestec u Prahy, Czech Republic

**Keywords:** FIB-SEM, CLEM, invadopodia, MT1-MMP, invasiveness

## Abstract

Cancer cell invasion through tissue barriers is the intrinsic feature of metastasis, the most life-threatening aspect of cancer. Detailed observation and analysis of cancer cell behaviour in a 3D environment is essential for a full understanding of the mechanisms of cancer cell invasion. The inherent limits of optical microscopy resolution do not allow to for in-depth observation of intracellular structures, such as invadopodia of invading cancer cells. The required resolution can be achieved using electron microscopy techniques such as FIB-SEM. However, visualising cells in a 3D matrix using FIB-SEM is challenging due to difficulties with localisation of a specific cell deep within the resin block. We have developed a new protocol based on the near-infrared branding (NIRB) procedure that extends the pattern from the surface grid deep inside the resin. This 3D burned pattern allows for precise trimming followed by targeted 3D FIB-SEM. Here we present detailed 3D CLEM results combining confocal and FIB-SEM imaging of cancer cell invadopodia that extend deep into the collagen meshwork.

## 1. Introduction

Cellular migration and invasion through tissue barriers is a key ability of cells in a variety of both physiological and pathological scenarios, including migration of immune cells to sites of infection or dissemination of cancer cells during metastasis [1]. Cell motility and tissue invasion depends on a direct physical interaction of the cell with the surrounding environment. The actin microfilaments attach to specialised sites at the plasma membrane—integrin-based adhesions, which link cells to the surrounding extracellular matrix (ECM). Invadopodia are specialised cell-matrix contacts with an inherent ability to lyse ECM components [2]. The knowledge of detailed morphology of invadopodia in 2D came from experiments where cells were seeded typically on gelatin-coated glass cover slips and invadopodia were visible as puncta, as is widely accepted. Nevertheless, detailed description of the 3D structure of invadopodia is crucial for the understanding of cancer cell invasiveness. In a chemoinvasion transwell assay, the invadopodia are formed within matrigel-filled pores and exhibit a pseudopodia-like appearance [3]. The cultivation of cells in dense/complex fibrillar 3D collagen (such as a thick layer of matrigel, skin-based matrix, high-density fibrillar collagen (HDFC) etc.) usually leads to a formation of a phenotype that can be described as a protruding base from which numerous thin filopodia-like filaments extend, as it was shown in original findings [4,5]. This model is supported by observations that Tks5 protein, a prominent invadopodial marker, is accumulated in the protruding base [6,7]. A similar structure of invadopodia was visualised by fluorescent microscopy in 3D collagen hanging droplet spheroids [7] and using HDFC collagen [7].

In order to study the 3D ultrastructure of cellular invadopodia, we developed a new Correlative Light and Electron Microscopy (CLEM) approach combining laser scanning confocal microscopy equipped with visible and near infrared lasers and with high numerical aperture and long working distance optics together with FIB-SEM tomography.

Correlative Light and Electron Microscopy (CLEM) is already a well-established method that combines two microscopy approaches for a deeper understanding of the same sample [8]. At cellular level, this allows for sequential study of molecular function by live cell light (fluorescence) microscopy (LM), where a molecule of interest is tagged specifically by a fluorescent protein, which is followed by the visualisation of the cellular ultrastructure in the region of interest by electron microscopy (EM).

The main struggle of the CLEM approach is the localisation of the same region of interest in the EM after LM imaging, which requires the introduction of fiducial markers visible in both microscopic modalities. Cells cultured in a monolayer require fiducials only in a single plane (XY axis). This can be reproducibly achieved using the gridded coverslip that enables embedding of the pattern into the resin [9]. However, the precise localisation of an LM-targeted cell in 3D (XYZ axis) is more problematic due to the technical limitations (the need for some type of serial sectioning and/or limited field of view) and sample destructive nature (cutting, milling) of electron microscopy imaging in the axial direction. Particularly, for FIB-SEM acquisition, using the trench approach as on our FEI Helios NanoLab 660 G3 UC microscope, the axial depth for high-quality acquisition is limited to around 40 µm. This means that the resin-embedded sample must be trimmed with micrometer precision to bring the selected volume of interest just underneath the resin block surface. To overcome these obstacles, Bishop et al. introduced near-infrared branding (NIRB) that enabled the induction of micrometer precise fiducials directly into the tissue before its embedding into a resin [10]. In brain tissue, NIRB was successfully used for localisation and correlation of targeted structures and cells in 3D. Recently, 3D localisation of the region of interest using only internal fiducials in brain tissue has been described [11]. However, the sample of interest in this scope is a combination of the above mentioned: a monolayer of cells grown on a thick layer of collagen. Thus, the aim of this scope was to design a CLEM experimental procedure for a specific sample (fibrosarcoma cells cultivated on a 50–200 μm thick layer of collagen) that inherently does not contain enough spatial clues for correlation. Therefore, we applied the known procedure of NIRB and applied it in a, to our knowledge not yet implemented, procedure in which fiducial markers for correlation were induced into resin-embedded samples for EM microscopy. This allowed us to use ultramicrotomy trimming efficiently and repeatedly for precise exposition of the targeted cell before FIB-SEM imaging.

## 2. Results

### 2.1. Invadopodia Were Resolved by Live-Cell Imaging

**Figure 1:**
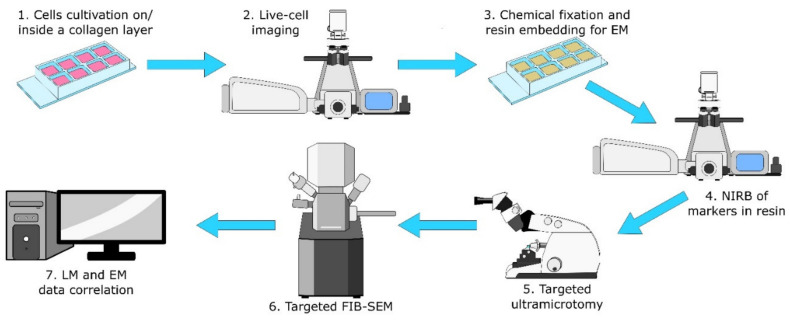
Scheme of the protocol. (**1**) Cells cultivation on/inside a collagen layer in eight-well chambers. (**2**) Live-cell confocal imaging of the target cell. (**3**) Chemical fixation and resin embedding for EM. (**4**) In-resin NIRB of fiducial markers for XY and Z location of the target cell. (**5**) Targeted ultramicrotomy for target cell exposition for FIB-SEM. (**6**) FIB-SEM data acquisition of the target cell. (**7**) FIB-SEM data alignment and correlation of LM and EM data. The same eight-well plate was used for cell growth, live-cell imaging, EM sample preparation and induction of markers (steps 1–4). Therefore, position of the objective lens relative to each well after live-cell imaging could be used for rough estimation of XY coordinates of the target cell prior to NIRB of markers (step 4).

The protocol consists of seven steps that are depicted in Figure 1 (and a detailed step-by-step form of the protocol is added as a Protocol S1 in the Supplementary Materials files). Readers should note the usage of the same eight-well plate for the first four steps for easy workflow and the target cell localisation for the NIRB step. Initially we tried to use commercial gridded coverslips for reproducible XY navigation within the sample. Unfortunately these gridded coverslips are not suitable for the collagen layer preparation (size, fragility). Instead we adopted the approach of hand-made scratches using a diamond-tipped scriber combined with precise sample and stage positioning for easier XY navigation.

**Figure 2:**
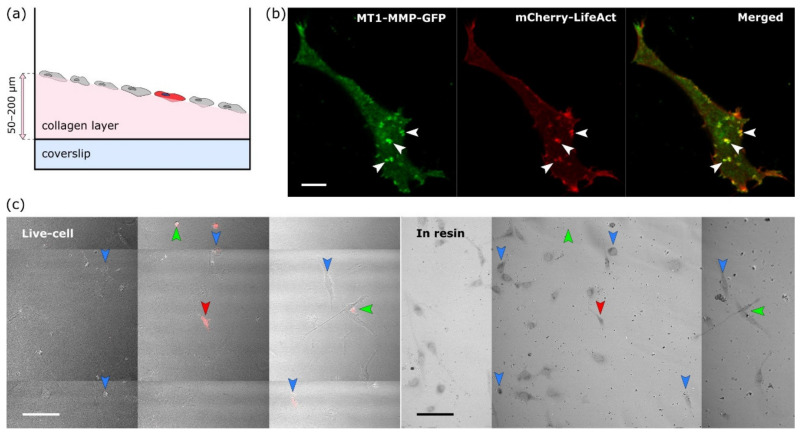
Live-cell imaging and resin embedding. (**a**) Scheme of a single well with varying depth of collagen. Target cell is marked in red. (**b**) Single plane of a Z-stack from live-cell imaging of a HT1080 fibrosarcoma cell. Examples of successfully resolved invadopodia are marked by arrowheads. Scale bar 10 μm. (**c**) Comparison of the overview tile scans after live-cell imaging (left) and resin embedding for EM (right). The majority of the cells (a couple of examples are marked by blue arrowheads) and the target cell (red arrowhead) were fixed successfully. Cells washed away in the process are marked by green arrowheads. Scale bars 100 μm.

HT1080 cells transiently transfected with MT1-MMP-GFP and Life-Act-mCherry were grown on a thick layer of HDFC collagen [12]. We used HDFC collagen because it mimics a dense fibrillar collagen matrix that becomes cross-linked in the course of tumorigenesis and that leads to increased number of invadopodia in cancer cells. Cross-linked HDFC also increases total numbers of invadopodia per cell [12]. To visualise cells located on up to 200 μm thick HDFC collagen (scheme in Figure 2a) and simultaneously gaining maximum available resolution, we acquired live-cell images via 40× W, 1.1 NA objective with a working distance of 620 μm. Z-stacks were acquired for no more than 2 min and with a minimal laser power, using a single photon counting detection mode, to preserve the physiological structure of invadopodia. With these limitations and after improving signal to noise ratio by deconvolution, we were able to resolve the structures of interest in the target cell by both staining methods—Life-Act-mCherry actin staining and MT1-MMP-GFP (Figure 2b, examples of resolved invadopodia are marked by arrowheads).

### 2.2. The Target Cell Was Localised after Resin Embedding and Its Position Marked by NIRB

Immediately after fluorescence image acquisition, the samples were chemically fixed directly on the microscope within seconds. For the approximate localisation of the target cell in each well, Z position of the objective relative to the well surface, XY position of the stage and the position of the samples on the stage were carefully marked and then the samples were moved onto ice. After EM sample preparation, the target cell, already embedded in the resin, was localised on the same confocal microscope by placing the well into the identical position on the stage, moving the stage to stored XY coordinates and the objective to the same relative Z position. Large area image obtained by a tile scan acquisition in transmitted light was then compared to one acquired during live cell imaging. Due to the specific cellular pattern, the target cell in the resin could be easily localised (Figure 2c, right). This process was also a good way to check that the immediate chemical fixation had not induced major morphological changes (compare left and right images in Figure 2c).

**Figure 3:**
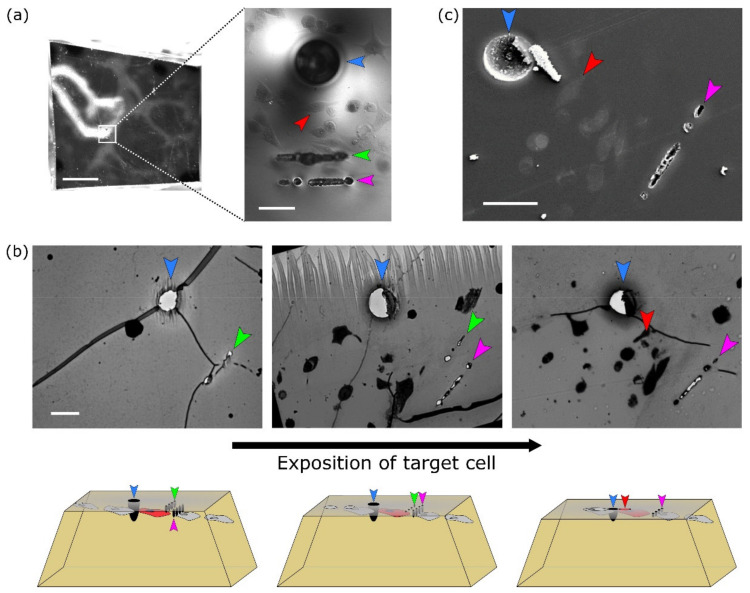
Ultramicrotomy guided by NIRB markers was used for exposition of target cell prior to FIB-SEM imaging. (**a**) Left: Top surface of resin block pyramid before ultramicrotomy trimming; Right: Magnified view of region of interest showing target cell (red arrowhead) and markers for orientation in XY (big bubble, blue arrowhead) and Z-lines (two sets of markers with different depths in Z, green and magenta arrowheads). Readers should note the out of focus green line located closer to the trimmed surface. Scale bar 1 mm (left), 50 μm (right). (**b**) Top row: Toluidine blue-stained ultramicrotomy sections show stepwise Z-line marker appearance. Note the simultaneous exposure of the target cell and disappearance of the green *Z*-axis marker on the right-most slice. Bottom row: Illustration of target cell exposition. Upward pointing magenta arrowhead in the leftmost image marks a not yet exposed Z-line. Arrowhead colour code same as (**a**). Scale bar 50 μm. (**c**) Exposed target cell and both XY and Z-line markers as seen by SEM prior to milling of the trench for FIB-SEM imaging. Arrowhead colour code as (**a**). Scale bar 50 μm.

Once the cell was localised in resin, fiducial markers for ultramicrotomy and FIB-SEM were introduced by NIRB. For easier trimming workflow for FIB-SEM, two types of NIRB markers were induced into resin:

(a) Big bubble marker(s) for macro-orientation in the XY; easily visible with the ultramicrotome optics (blue arrowhead in Figure 3).
(b) Two lines of smaller bubble Z-markers at different depths that were later used for orientation along Z axis, thus allowing for a precise termination of ultramicrotomy block trimming (green and magenta arrowheads in Figure 3). The first line of Z-markers was placed in the focal plane of the target cell, the second line of Z-markers was placed 10 μm above the target cell. The Z-marker line marked green in Figure 3a is located closer to the surface of the resin block, therefore is out of focus on the given image.

After each step of NIRB marker induction, the resulted markers were inspected in transmitted light.

### 2.3. Optimalisation of In-Resin Bubble Formation

The bubble formation was not perfectly reproducible using the same microscope setup for different samples. It was noted that the formation of bubbles depended on the age and the batch of resin. Simultaneously, changes in the thickness of the collagen layer led to the necessity for variances in settings of the NIRB procedure as well. To overcome resin age-dependent changes, bubbles were always introduced into freshly polymerised resin, within the first day after polymerisation. Differences in batch and depth meant we had to test bubble induction each time prior to forming final marker bubbles. First, the thickness of collagen layer was measured (difference between Z positions of coverslip and cells). Then, the sample was moved to focus into the corner of the well to perform testing of bubble creation far away from the target cell. After optimisation of the needed NIR laser power and time (scanning speed), the sample was moved back to focus near the cell of interest to induce the final marker bubbles. It is recommended to always carry out the test bubbles first, as improper settings for bubble induction could easily lead to the destruction of the cell of interest.

### 2.4. Targeted Ultramicrotomy Successfully Exposed the Target Cell for FIB-SEM Imaging

After NIRB, the well-plate was removed, leaving a pure resin block with a flat bottom and bubble markers inside. Taking into account the rather flat geometry of cells grown on the collagen layer, sample trimming was be ideally to be performed from the bottom through the collagen layer or from the top through the thick empty resin. Initially, the bottom-trimming approach was used, which seems to be more natural and is easier to perform. Our first trials discovered that approaching the target cell from the collagen layer possess a high risk of missing (removing) some invadopodia protruding deep inside the collagen, which are not visible in optical microscopy. Based on this experience, the top-trimming approach was used for the rest of the experiments.

Before the target cell exposure by ultramicrotomy, the resin block was downsized to a small cube and later trimmed into a small pyramid around the target cell (Figure 3a, left). For orientation under the stereomicroscope, big bubble markers were used. While trimming, the big XY marker(s) were visible with the ultramicrotome optics, while cells and *Z*-axis markers had to be visualised by staining sections with toluidine blue (Figure 3b, top). The *Z*-axis markers, labeled green and magenta in the Figure 3, were 10 µm apart in the Z-direction. At first, the top of the pyramid was trimmed in large steps (thickness of slices 1 μm). Once the first line of *Z*-axis markers emerged (Figure 3b, left, green arrowhead), trimming steps were decreased to 0.2 μm per slice. The appearance of the next Z-line on the slices (Figure 3b, middle, magenta arrowhead) marked a closer distance to the cell of interest. The targeted cell was exposed on the block surface just as the green Z-line disappeared (Figure 3b, right). At this point, the targeted ultramicrotomy trimming was complete.

### 2.5. FIB-SEM Data Acquisition

The target cell was localised by comparing images from optical microscopy with overview SEM images of secondary electrons from the trimmed block surface (Figure 3c). Exposed NIRB markers are easily visible on the surface of the resin block (Figure 3c, blue arrowhead for XY marker and magenta arrowhead for Z marker line). Therefore, the target cell can be readily and unambiguously localised for FIB-SEM imaging (Figure 3c, red arrowhead).

FIB-SEM is a time-consuming and rather expensive technology. Instead of using a brute-force approach, which means acquisition of the whole cell with the best possible sampling and long pixel dwell times, resulting in weeks of acquisition per cell giving many hundreds of GB of data, we optimised the acquisition procedure to shorten the acquisition while giving sufficient data quality. Firstly, based on the fluorescence signal in live-cell images, the location of invadopodia was estimated in the resin block, so that only the relevant part of the cell needed to be imaged. Secondly, we tested different acquisition settings of pixel dwell time, pixel size and slice thickness to find a compromise between data quality and acquisition speed. Data quality was assessed by the success of automated image segmentation routines and visual impression for human inspection-based conclusions. The obtained compromise between quality and measurement time was 20 µs pixel dwell time, 6 nm pixel size and 20 nm slice thickness for our setup and samples. Next, the image size was adjusted during the acquisition to match the actual cell size in the slice. This means a larger image area in the middle of the cell, and smaller at the edges. Lastly, the produced data were continuously controlled and the acquisition was realigned in case of technical issues or stopped in case the selected sample volume turned out to not be biologically relevant. Altogether, seven datasets with complete workflow from live-cell imaging to at least partial FIB-SEM acquisition were obtained.

### 2.6. 3D Reconstruction of Invadopodia from FIB-SEM Data

The visual inspection of individual FIB-SEM slices identified the best dataset for further data processing. Confocal fluorescence datasets were deconvoluted, FIB-SEM data were denoised, aligned and registered with the fluorescence data. In the aligned and denoised 3D FIB-SEM dataset, plasma membrane, marking the cell periphery, of the selected invadopodium and the collagen fibres that are in contact or in close vicinity of the cell were segmented using a combination of automated and manual tracking.

**Figure 4:**
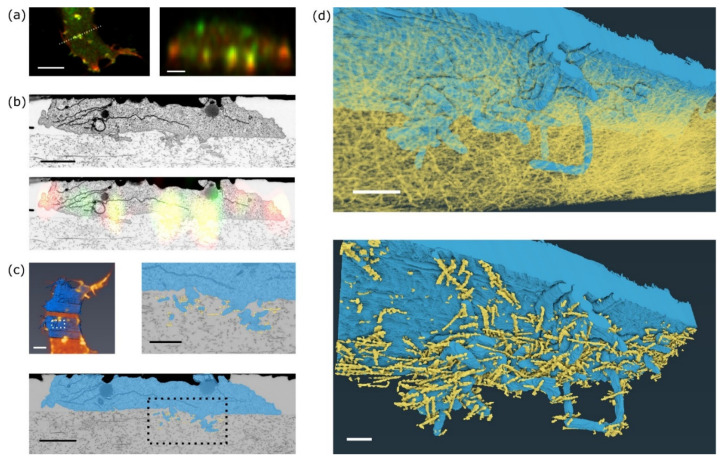
Data correlation and segmentation of invadopodia. (**a**) Position (left) and single xz slice (right) used for correlation between EM and LM data. Scale bars 10 µm (left), 2 µm (right). (**b**) Top: EM slice from FIB-SEM used for EM and FM correlation. Bottom: Overlay of LM and EM single slice showing overlap between invadopodia resolved in detail (EM) and localisation of MT1-MMP-GFP signal from live-cell imaging. Scale bar 2 µm. (**c**) Top left: 3D overlay of FIB-SEM data after alignment and segmentation (in blue) with actin-fluorescence (orange). Top right: Detailed view of segmentation of selected invadopodia (blue) growing into collagen fibres in direct contact with them, shown in yellow. Bottom: Overview of shape segmentation in a single slice from FIB-SEM. Dotted box marks area of detailed view shown in Top right. Scale bar 5 µm (top left), 1 μm (top right), 2 µm (bottom). (**d**) 3D projection of a detailed segmented invadopodium (cell shape in blue). Top: invadopodium (blue) inside a collagen meshwork (yellow). Bottom: collagen fibres in direct contact with cellular surface (yellow). Scale bar 1 μm (top), 500 nm (bottom).

In fluorescence images, invadopodia are visible as clusters of F-actin and MT1-MMP signals that protrude into the collagen matrix (Figure 4a). Registration of a single fluorescence slice in XZ of MT1-MMP-GFP and mCherry-LifeAct signal and a single slice from FIB-SEM shows localisation of the signal predominantly in cellular protrusions that extend into the collagen gel (Figure 4a,b). Consistently with our previous observations in acellular dermis [5], FIB-SEM images show invadopodia consisting of a base and several thin filopodia-like filaments extending deep into the collagen meshwork (Figure 4b, Supplementary Materials Video S1). The correlation of the segmented whole cell images between live cell fluorescence acquisition and FIB-SEM indicates that the cell shape was well preserved during EM sample preparation (Figure 4c, top left). A very similar structure of invadopodia was also visualised using an MDA-MB-231 cell (Supplementary Materials Figure S1 and Video S2).

3D reconstruction of the invadopodium and the surrounding collagen fibres was performed to reveal the mutual interconnections (Figure 4d, top). Fibres that are in direct contact with the invadopodium are highlighted in Figure 4d, bottom, and Supplementary Materials Video S3. Closer inspection of the FIB-SEM images reveals that collagen fibres are not only touching, but also going through cellular protrusions and these protrusions are wrapped around the fibres. Such wrapping was also detected previously in vivo by DHM microscopy [13].

## 3. Discussion

Correlative Light and Electron Microscopy (CLEM) by a combination of two complementary microscopy approaches enables enhanced visualisation and a deeper functional understanding of biological samples. As acquiring large volumes (brute-force approach), on the order of cubic millimetre, with high number of several nanometres of isotropic resolution, is and will be technically challenging, time-consuming (months to years) and producing enormous amount of data (hundreds of TB and more) [14]; the only practical way for obtaining ultrastructural details of rare events is to precisely target the region of interest in the EM sample based on previous live cell optical microscopy observation. However, for samples in a 3D environment, the precise localisation of the same region of interest in the EM after LM imaging is problematic. Our protocol is designed for cells growing fully in 3D; nevertheless, we took advantage of HDFC (high density fibrillar collagen) that mimics tumour desmoplastic collagen and inherently induces invadopodia formation [12]. Thus, cells grow in a semi-3D environment with an average distance from the glass coverslip ranging between 50–200 μm. We used invasive fibrosarcoma cells HT1080 transiently transfected with MT1-MMP-GFP as an integral membrane invadopodia marker and Life-Act-mCherry as an actin cytoskeleton marker. Both MT1-MMP and actin are enriched in invadopodia [15].

Finding the same region of interest in 3D CLEM experiments in all used imaging modalities is the key step for successful correlation of the obtained information. As live cell imaging always precedes electron microscopy acquisition, the question is how to be efficient in finding the target under the electron microscope. The newest development in cryo-CLEM allows the vitrified sample to be visualised, using fluorescence and SEM within one instrument, without the need of its transfer [16]. Cryo-CLEM is suitable for high resolution imaging of unstained samples, but so far lacks the ability of large volume imaging. Traditional in resin-embedded and heavy metal stained samples are thus still the mainstream for volumetric EM analysis. The optimal situation would be if the EM sample processing preserves the fluorescence signal [17], and the resin-embedded sample can thus be explored by both fluorescence and EM at the same instrument, which would provide the ultimate signal registration. Unfortunately, the choice of dyes and their performance for in-resin fluorescence is still very limited.

There are multiple approaches for volumetric EM, for example Serial Array Tomography, Serial Block Face SEM or FIB-SEM. Despite slightly different ways of obtaining 3D data, having 3D markers imprinted in the sample would make the acquisition faster and more precise for all of them. Here we focused on application of 3D markers for FIB-SEM approach.

The standard procedure for correlation of images between optical and electron microscopy using in-resin samples is to use special gridded coverslips. This approach works well for adherent cells but fails for targeting cells located further away from the coverslip, for example for cells grown on a thick layer of collagen. The reason is that (a) a cell deep inside the resin cannot be localised by an overview SEM, and (b) the depth of the milled trench for high quality FIB-SEM is limited. To ensure the cell is in the working distance of FIB-SEM, it is necessary to trim away the surface layer of resin up to the targeted cell. Unfortunately, this also removes the imprinted grid necessary for the cell localisation. We have shown that modified NIRB workflow that extends the pattern from the surface grid deep inside the resin block by using IR laser-induced bubbles overcomes this limitation. This 3D burned bubble pattern allows for precise trimming followed by targeted 3D FIB-SEM.

From a technical point of view, any inverted laser scanning confocal microscope equipped with NIR pulsed laser for two-photon excitation can be used for both live cell imaging and subsequent NIRB. Care must be taken to optimise the NIRB settings for each sample, as parameters such as the age of the resin, depth and amount of heavy atom staining may influence the branding.

Potentially, this procedure could be used for straightforward cell localisation and correlation in CLEM procedures using TEM grids, due to ever-present markers that would directly overcome issues such as rotation and accidental flipping of the ultramicrotomy slices. However, samples after osmification are not always transparent to allow visual localisation using light microscopy, as was the case here (Figure 3c).

Localised degradation of the surrounding matrix is seen at invadopodia in a variety of aggressive carcinoma cells, suggesting that these membrane-associated cellular structures play a central role in mediating polarised migration in cells that cross tissue boundaries [2]. MT1-MMP-mediated cell invasion has been implicated in different disease processes, including inflammation, atherosclerosis, rheumatoid arthritis, cancer invasion, and metastasis (reviewed in [18,19]). In agreement with our previous observations, we now show in higher detail that the mature invadopodia consist of a thick base from which thin protrusions extend deep into the surrounding collagen mesh-work [5]. It was reported [5,20] that the main place of collagen degradation is not at invadopodia/pseudopodia tips, but close to the cell body. Detailed slide after slide observation revealed that collagen fibres are not only in contact with the cell membrane, but also that the membrane is partially wrapped around the fibres. Wrapping of cell protrusion around collagen fibres was also detected by a digital holographic microscopy [13]. We also detected a membrane system and vesicles in those areas. Based on our recent data, limited by the resolution of the light microscopy system used, we cannot determine whether these vesicles bear ECM-lytic enzymes; however, if they do (as it was reported in [21]), this could facilitate the concentration of collagen fibres at sites where degrading enzymes are deposited, and it might represent the main place where fibres are cleaved. Our data might represent a 3D view of how this is organised.

Even though imaging cells growing on 2D cover slips can provide valuable information, it is important to observe cancer cell behaviour in a 3D environment for a full understanding of the mechanisms of cancer cell invasion. Here we presented detailed CLEM protocol combining confocal and FIB-SEM imaging of cancer cell invadopodia in 3D and located far from the Ibidi bottom coverslip. Our approach is suitable for various applications of cells growing inside a gel or for a piece of tissue placed inside a well.

## 4. Materials and Methods

### 4.1. Cell Culture, Transfection and HDFC Matrix

Human fibrosarcoma cells HT1080 and human breast adenocarcinoma cells MDA-MB-231 were routinely cultured in standard conditions (37 °C, humidified atmosphere with 5% CO_2_) in full DMEM medium (Life Technologies, Waltham, MA, USA) with 4.5 g/L L-glucose, L-glutamine, and pyruvate, supplemented with 10% fetal bovine serum (Merck KGaA (Sigma-Aldrich), Darmstadt, Germany) and 0.1% ciprofloxacin (Sigma-Aldrich). Cell transfections were carried out using Polyethylenimine-PEI (Sigma-Aldrich) according to the manufacturers’ protocols. MT1-MMP-GFP was a gift from prof. S. Courtneidge.

High density fibrillar collagen was prepared according to [12]. Briefly, glass or polymer bottom coverslips of µ-Slide 8 Well (Ibidi, Gräfelfing, Germany) were coated with Poly_D-Lysine ( Merck KGaA (Milipore), Darmstadt, Germany) for 20 min at RT so that only a circular area in the middle was coated. After a brief wash with PBS, 2% glutaraldehyde (Merck KGaA (Sigma-Aldrich), Darmstadt, Germany) was added to the same area for 10 min and washed three times with PBS. Collagen (2 mg/mL) was prepared on ice by mixing collagen (prepared from rat tails, stock 4 mg/mL) with a buffered solution (final concentration composed of 1× DMEM, 0.375% NaHCO3, 15 mM Hepes) and neutralised on ice by 1 M NaOH. The thin layer of collagen was allowed to polymerise into a fibrillar meshwork at 37 °C for 30 min. The µ-Slides were centrifuged at 3500× *g* for 20 min to flatten the collagen meshwork. To stabilise the HDFC matrices, centrifuged collagen matrices were fixed with 4% paraformaldehyde and 5% sucrose in PBS for 20 min, washed three times with PBS, and blocked with DMEM (supplemented with 20% FBS) overnight at RT.

### 4.2. Live-Cell Imaging

Live-cell images were acquired using a scanning confocal microscope (Carl Zeiss LSM 880 NLO; Carl-Zeiss, Oberkochen, Germany) equipped with 40× W, 1.1 NA objective (WD 620 μm), 32-channels GaAsP PMT spectral detector in photon counting mode for fluorescence detection (excitation by 488 nm and 561 nm) and transmitted light detector LSM T-PMT (used with 561 nm excitation). Acquisition of the target cell Z-stack was followed by acquiring a tile scan overview data (excitation 561 nm for both fluorescence and transmitted light images). At the end of image acquisition in each well, XY position of the stage and the position of the sample on the stage were carefully marked for cell localisation after fixation and EM sample preparation.

### 4.3. Live-Cell Imaging Fixation and Embedding for FIB-SEM Imaging

After confocal imaging, cells were immediately fixed on-microscope by a mixture of ice cold 2.5% glutaraldehyde and 1% formaldehyde in a fixation buffer (0.1 M sodium cacodylate, pH 7.2) and placed on ice for 1 h. Cells were then washed by the fixation buffer (3×, 5 min), post-fixed in reduced 1% OsO_4_ with 1.5% K(FeCN)_6_, then in 1% OsO_4_ (both in fixation buffer, 30 min, on ice), washed with the fixation buffer, distilled H_2_O, and contrasted with 1% uranyl acetate in H_2_O (30 min, no light, room temperature). After rinsing with distilled H_2_O, samples were passed through dehydration steps with increasing concentrations of ethanol (30%, 50%, 80%, 95%, 100%, 2 min each, except 5 min in 100%). At last, samples were embedded in Embed812 (polymerised for 72 h at 60 °C). All the steps were performed in the 8-well plate used for live-cell imaging [22].

### 4.4. Cell Localisation in Resin and Formation of Fiducial Markers by NIRB

The target cells were localised after EM embedding by placing the sample into identical position on the microscope stage and moving the stage into stored coordinates as it was during live cell imaging. Tile scans acquired after live-cell imaging were then compared with in-resin cell pattern. Fiducial markers for ultramicrotomy and FIB-SEM were then introduced by NIRB using Carl Zeiss LSM 880 NLO confocal microscope equipped with a pulsed Ti:Sapphire femtosecond laser Chameleon Ultra II (Coherent, Santa Clara, CA, USA), and 25× Imm, 0.8 NA objective with water immersion. NIRB markers were induced by the Ti:Sapphire laser tuned to 810 nm with laser power around 70 mW. The optimal laser power was adjusted for each sample. Big bubble marker(s) were induced by focusing the IR laser to a single point for 1 s and line markers were induced by scanning a 100 μm-long line repeatedly for ~10 s (single line scanning time 509 µs, 20,000× repeats, 2 Z-planes). After each step of the NIRB marker induction, resulting marker(s) were inspected using transmitted light detector.

### 4.5. Targeted Ultramicrotomy

The block of resin from each well was downsized for ultramicrotomy, attached to the top of a blank resin block with acrylic glue and trimmed to a small pyramid around the target cells guided by the big bubble markers visible under the stereomicroscope. Leica UC7 ultramicrotome equipped with a glass knife was used for trimming. While trimming, the big XY marker(s) were visible with the ultramicrotome optics, cells and *Z*-axis line markers were visualised by staining sections with toluidine blue. Upon reaching the big NIRB markers, thickness of slices was reduced from 1 μm to 0.2 μm, which allowed for precise trimming completion and the target cell exposure for FIB-SEM.

### 4.6. FIB-SEM Imaging

FIB-SEM data were acquired using FEI Helios NanoLab 660 G3 UC. For imaging, the sample was mounted on a regular SEM stub using conductive carbon and coated with 25 nm of platinum (using High Vacuum Coater, Leica ACE600). The target cell was localised by comparing images from optical microscopy with overview SEM of secondary electrons signal from the trimmed block surface (30 kV, 0.8 nA, Everhart–Thornley detector). On the top of a localised target cell, a protective layer of platinum (1000 nm) was deposited using a single gas injection system. Around this area, the large trench was milled (21 nA, 30 kV), and subsequently polished (0.79 nA, 30 kV, thickness of slices 20 nm). SEM images were acquired at 2 kV, 0.2 nA, using the In-Column backscattered electron detector (ICD) with pixel size 6 nm and a pixel dwell time 20 µs. The size (number of pixels) of acquisition area was adjusted to the size of the cell.

The whole datasets were collected using the Thermo Scientific Auto Slice and View 4 (AS and V4) software (net acquisition time was 34 h). The data acquisition process is automatic but requires human supervision as instrument errors do occur. Occasionally some 3D datasets miss several slides due to a hardware error during data acquisition.

### 4.7. Data Analysis

Acquired fluorescence data in both channels were deconvolved using an automatic procedure in the Huygens 19.10 software with signal to noise ratio set to 3 and an inbuilt, automatically generated point spread function for the used objective and excitation wavelength.

The FIB-SEM datasets were denoised by denoise.ai plugin in NIS-Elements and further processed with Amira Software 6.2. After the alignment of 2D slices, complete 3D FIB-SEM data were rotated into the same orientation as the fluorescence Z-stack and manually registered. Overlay of a single-plane FIB-SEM electrogram with deconvolved fluorescence data was performed in the ec-CLEM plugin for Icy.

Manual tracking of organelles and the cell body was performed using the Amira segmentation module. The masks of the cell body in a region of interest were manually perfected to better fit the shape of the cell and the invadopodia. Collagen fibres were extracted from aligned and denoised EM data using Python (OpenCV, Palo Alto, CA, USA; and skimage). To observe behaviour of collagen fibres near the body and invadopodia of the cell, only collagen fibres in contact with the cell were extracted. The extraction is described below.

All images $I_{i}$ were processed by AdaptiveThreshold (with parameters adaptiveMethod = ADAPTIVE_THRESH_MEAN_C, blockSize = 13, C = 6) to create segmentation masks $M_{i}$ of the collagen fibres, where i was the position of a slice in the Z-stack. Where the segmentation mask $C_{i}$ was a set of all segmented pixels of the cell body and mask $M_{i}$ was a set of all segmented pixels of the collagen fibres, set subtraction had been applied to clear any undesirable objects inside the cell body (and the cell body itself), so that collagen masks $M_{i}$ did not contain any pixels from their corresponding cell masks $C_{i}$.

Applying the following Equation (1) to each image slice *i*, we defined individual fibres $T_{p,i}$ as all pixels q, such that there existed path a between p and q in the segmentation mask $M_{i}$:

(1) $$T_{p,i\;}=\;\{q\;|\;q\;\in M_{i},\;\exists \;a\;=\;\left(q,\;c_{1},\ldots ,\;c_{n},\;p\right),\;a_{j}\in M_{i},\;a_{j+1}\in N_{a_{j}}\}$$

where $N_{r}$ is 8-connected neighborhood of the pixel r. Thus, a set for each pixel p with all the pixels was belonging to the same collagen fibre as pixel p was created. Further, we consider only set S of fibres $T_{p,i}$ with more than five pixels, i.e., $S=\{T_{p,i}:\;|T_{p,i}|\;>\;5\}$.

As only collagen fibres neighbouring the cell were of interest to us, we further refined the set S to contain only fibres in contact with the segmentation mask $C_{i}$, ie. $S\prime =\{T_{p,\;i}\;|\;\exists q\;\in T_{p,\;i},\;r\in \;C_{i,\;}q\in N_{r}\}$. We enriched the set S′ by iteratively adding overlapping fibres in the neighbouring Z-plane. The iterative process was defined with an initial set $S\prime_{1}=S\prime$, and k-th iteration of the enrichment defined as $S\prime_{k}=S\prime_{k-1}\;\cup \{T_{p,\;i}\;|\;T_{p,i\;}\cap T_{q,i\pm 1\;}\neq \;\emptyset ,\;T_{q,i\pm 1}\in S\prime_{k-1}\}$, where $T_{q,\;i\pm 1}$ represented either $T_{q,\;i-1}$ or $T_{q,\;i+1}$. The enrichment ensured the continuity of a collagen fibre across more planes, as long as there had been contact in at least one plane, even if no contact between the fibre and the cell body was present in any of the following Z-planes.

Resulting collagen masks were exported as binary images, as well as EM data with cell and collagen masks overlayed to showcase the results in 2D. The resulting masks of the cell and collagen were converted to labels and visualised in Amira as 3D projection of the segmented cell and collagen.

## Data Availability

Not applicable.

## Supplementary Materials

The following are available online at https://www.mdpi.com/article/10.3390/ijms22157805/s1, Video S1: Electron microscopy slices from FIB-SEM used for EM and FM correlation; Video S2: Electron microscopy slices from FIB-SEM (MDA-MB-231 cell); Video S3: 3D projection of invadopodium; Figure S1: Invadopodia of MDA-MB-231 cell visualised by fluorescent and electron microscopy; Protocol S1: A detailed step-by-step protocol.
