# Supplementary material for: Invadopodia Structure in 3D Environment Resolved by Near-Infrared Branding Protocol Combining Correlative Confocal and FIB-SEM Microscopy
Source: Int J Mol Sci. 2021 Jul 21;22(15):7805. doi: 10.3390/ijms22157805 (PMC8346040; doi:10.3390/ijms22157805)
Supplement: Supplementary file 1 [file ijms-22-07805-s001.zip › Figure S1 rev.pdf]

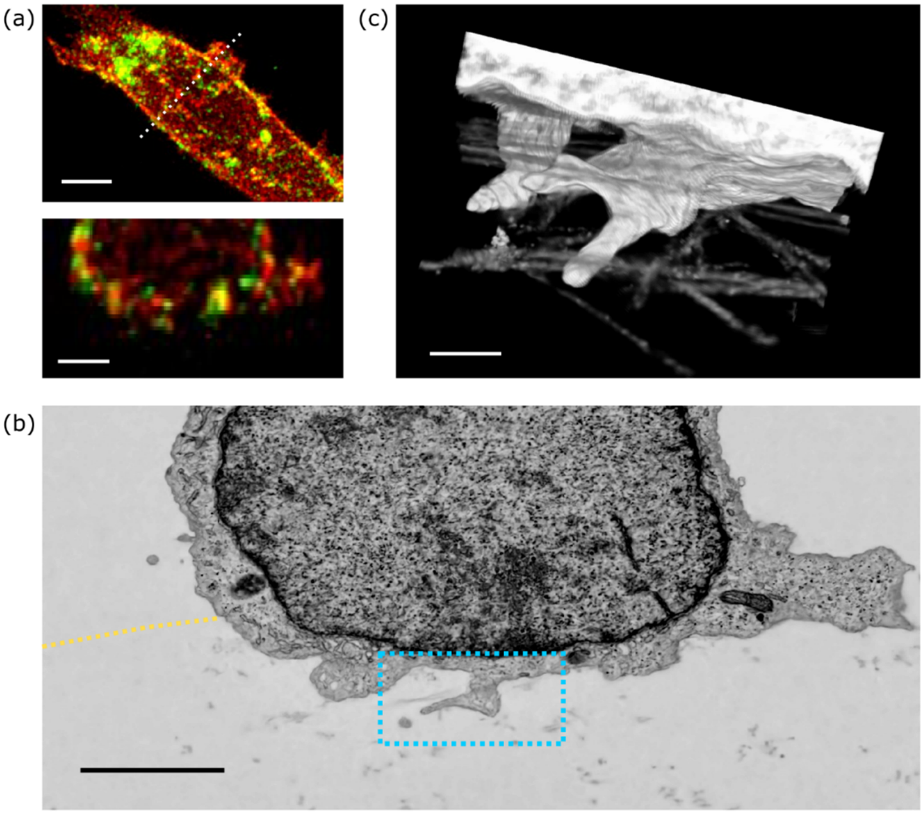

Figure S1. Invadopodia of MDA-MB-231 cell visualised by fluorescent and electron microscopy. a) Fluorescence microscopy images of MDA-MB-231 cell line (green: MT1-MMP-GFP, red: mCherry-LifeAct). **Top:** A cell body with marked position of a single xz slice. **Bottom:** Single xz slice from the same position of the cell body as FIB-SEM slice in (b). Scale bars 5 µm (top), 2 µm (bottom). **b)** Single EM slice of MDA-MB-231 corresponding to the LM xz slice in (a). Blue square represents position of the 3D rendered region in (c). Yellow line represents a position of the top of a collagen layer. Scale bar 2 µm. **c)** 3D rendering of an invadopodium. EM dataset (101 slices with (b) slice in the center) was denoised using NIS-Elements denoise.ai, cropped in the area specified in (b), inverted, and rendered in 3D using ICY. Visualisation of collagen filaments was achieved by applying a manual threshold in an intensity histogram. Scale bar 0.5 µm.
