# Supplementary material for: Invadopodia Structure in 3D Environment Resolved by Near-Infrared Branding Protocol Combining Correlative Confocal and FIB-SEM Microscopy
Source: Int J Mol Sci. 2021 Jul 21;22(15):7805. doi: 10.3390/ijms22157805 (PMC8346040; doi:10.3390/ijms22157805)
Supplement: Supplementary file 1 [file ijms-22-07805-s001.zip › Protocol S1.pdf]

Protocol S1: A detailed step-by-step protocol for near-infrared branding procedure combining correlative confocal and FIB-SEM microscopy for 3D CLEM visualization of cells and cell structures deep inside a resin.

1. Collagen preparation

1. Preparation of collagen should be performed according to standard procedures. Alternatively, High density fibrillar collagen (HDFC), which is much more resistant to physical disruption during the long procedure, can be used.
2. For HDFC preparation (see [10] for more details) use the advantage of μ-Slide 8 Well (Ibidi), coat the bottom with Poly_D-Lysine (Millipore) for 20 min at RT so that only a circular area in the middle is coated.
3. After a brief wash with PBS, add 2 % glutaraldehyde (Sigma) to the same area for 10 min and wash three times with PBS.
4. Prepare collagen (2 mg/ml) on ice by mixing collagen (prepared from rat tails, stock 4 mg/ml) with Buffered solution (final concentration composed of 1x DMEM, 0.375% NaHCO3, 15 mM Hepes) and neutralize on ice by 1 M NaOH. Make a thin layer of collagen on pre-coated slides and allow the collagen to polymerize into a fibrillar meshwork at 37°C for 30 min.
5. Centrifuged the μ-Slides at 3,500 g for 20 min to flatten the collagen meshwork.
6. To stabilize the HDFC matrices, fix them with 4% paraformaldehyde and 5% sucrose in PBS for 20 min, wash three times with PBS, and block with DMEM (supplemented with 20% FBS) overnight at RT.
7. Seed cells on top of collages at least 24 hours before imaging.

2. Live-cell imaging

8. Make sure that selected microscope is equipped with an objective that combines high numerical aperture and long working distance.
9. Prepare fixation buffer (0.1M sodium cacodylate, pH 7.2) and fixation mix (2.5% glutaraldehyde and 1% formaldehyde in fixation buffer). Keep the fixation mix on ice.
10. Start live-cell imaging by finding cells of interest in each well and mark/store their XY coordinates. *If different microscopes will be used for live-cell imaging and NIRB (near-infrared branding), use smartphone camera to mark position of the objective below the well.*
11. Acquire live-cell images and overview tile-scans in each well.

3. Fixation and embedding for FIB-SEM imaging (perform all the steps in the 8-well plate)

12. Fix cells continuously in each well on-microscope immediately after imaging by removing the medium and adding the fixation mix.
13. After live-cell imaging, continue with fixation on ice for total time of 1 h.
14. Wash cells with fixation buffer (3x, 5 min) and post-fix them in reduced 1% OsO_4 with 1.5% $K_4[(FeCN)_6]$, then in 1% OsO_4 (both in fixation buffer, 30 min, on ice).
15. Wash cells with fixation buffer and dH_2O.
16. Contrast cells with 1% uranyl acetate in dH_2O (30 min, no light, room temperature).
17. Rinse cells by dH_2O and dehydrate the sample in ethanol series (increasing concentration: 30%, 50%, 80%, 95%, 100%, 2 min each, except 5 min in 100%).
18. Embed the sample in Embed812 and let it polymerize for 72 h at 60°C.

4. Cell localisation in resin and formation of fiducial markers by NIRB

19. Place the 8-well plate with resin embeded sample onto a microscope equipped with an IR laser.

20. Recall the stored XY positions of the stage. *If different microscopes are used, place the objective to the same position below the well using the smartphone images.*
21. Focus on the layer of cells and acquire an overview tile-scan. *Larger tile-scan might be required if different microscope is used because of the approximate XY starting position of the stage.*
22. To localise the target cell, compare the pattern and shapes of the cells in the in-resin overview tile-scan with the one acquired after live-cell imaging.
23. After target cell localisation, update the XY stage coordinates in the corresponding well.
24. Move stage to the corner of the well to perform an in-resing NIRB test.
25. Tune laser to desired wavelength and power (in our case 810 nm and approx. 70 mW) and start NIRB of a test XY-marker by focusing the IR laser into a single point for 1 s.
26. For good visibility of XY-markers under a stereomicroscope, repeat the marker induction and fine tune both the intensity and time interval of NIRB until the diameter of the big XY-marker is roughly 25-50 μm.
27. Move stage to the stored XY position of the target cell.
28. Induce 2-3 big XY-markers by focusing into a single spot with the fine tuned microscope setup. Make sure that the induction of markers will not destroy the targed cell.
29. Continue to make 2-3 Z-markers by NIRB for precise end of microtomy trimming:
    Focus onto the target cell and specify the position of the 1st Z-marker that is in same depth as the target cell. In our case, 100 μm long line that was scanned repeatedly with IR laser for approx. 10 s (single line canning time 509 μs, 20 000x repeats) with the same microscope setup as was used for XY-markers.
    More Z-markers can be introduced by moving objective up or down in a desired Z-direction and specifying their position next to the previous one(s).
30. Capture an overview image with all of the NIRB markers. Make sure to mark the distances between the Z-markers to aid with the orientation in the resin block while trimming.
31. Use permanent marker to mark the position of the target cell in-resin and repeat the induction of fiducial markers for the remaining wells.

5. Targeted ultramicrotomy

32. Disassemble 8-wells and downsize the resin block using a razor according to the marked position of the target cell.
33. Attach the downsized resin to the top of a blank resin block with acrylic glue and trim it to a small pyramid around the target cell under a stereomicroscope. Use the bigger XY markers for orientation.
34. Trim the pyramid with the ultramicrotome. Start with thick slices for faster workflow (1 μm in our case) and reduce the thickness upon reaching the XY markers (0.2 μm in our case).
35. Stain the thinner slices with toluidine blue to visualise Z-line markers and cells.
36. Stop the trimming process once the top of the target cell appears on the slices. If FIB-SEM dataset of the entire target cell is required, stop the trimming process upon reaching the Z-line markers located close to the target cell.

6. FIB-SEM imaging

37. Mount the sample on a regular SEM stub using conductive carbon and coat it with 25 nm of platinum.
38. Localise the target cell by comparing images from optical microscopy with overview SEM of secondary electrons signal from the trimmed block surface.
39. Deposit a protective layer of platinum on the top of the localised target cell.
40. Mill and polish the trench around this area.

41. Acquire FIB-SEM dataset.

4.7. Data analysis

Perform analysis and overlay of acquired data in preferred software.

In our case we combined Huygens 19.10 (deconvolution of fluorescence datasets), NIS-Elements plugin denoise.ai (EM data denoising), Amira 6.2 (alignment of FIB-SEM data, manual registration of LM and EM data, manual segmentation of EM data), ICY plugin ec-CLEM (overlay of a single EM and LM slice) and Python libraries OpenCV and skimage (extraction of the collagen fibers).

All the details are available in the Materials and Methods section.
